# Supplementary material for: Factors influencing healthcare provider respondent fatigue answering a globally administered in-app survey
Source: PeerJ. 2017 Sep 12;5:e3785. doi: 10.7717/peerj.3785 (PMC5600176; doi:10.7717/peerj.3785)
Supplement: Appendix S1 [file peerj-05-3785-s001.pdf]

Supplementary Appendix

Table of Contents

Table S1: Survey for collection of basic demographic information …………………………........... 2
Table S2: Survey for collection of information about sugammadex…………………….............. 3
Figure S1: Screenshot of the app ....………………………………………………………….., 4
Survalytics Detailed Description …………………………………………………………….... 5
Mobile Healthcare App Study JSON Document Schema ....……………………………..…… 8
Methodology for Calculation of App Use Frequency ....……………………………………….. 15
References ……………………………………….………………………………………18

Table S1: Survey for collection of basic demographic information from users of the app.

Question	Responses	
About how long have you been using the app (in months)? (Select 0 if you are a new user.)	Range: 0-100	
What is your level of medical training?	Physician: Attending/Consultant<br>Physician: Fellow/Resident/Registrar<br>Anesthesia Assistant (PA)<br>Nurse Anesthetist (CRNA)<br>Nurse (RN)<br>Technically Trained in Anesthesia<br>Anesthesia Technician<br>Student AA	Student Nurse Anesthetist<br>Medical Student<br>Paramedic/EMT<br>I am not a medical practictioner<br>Respiratory Therapist<br>Pharmacist<br>Other type of medical provider
What are your medical specialties?	Anesthesiology<br>Pain Medicine<br>Pediatric Anesthesiology<br>Cardiothoracic Anesthesiology<br>Obstetric Anesthesiology<br>Adult Critical Care	Pediatric Critical Care<br>Emergency Medicine<br>Pediatrics<br>Internal Medicine<br>Other
How many years have you been in practice? (Not counting years in training)	Range: 0-50	
Please rate the importance of the app to your practice:	Not Important At All<br>Of Little Importance<br>Of Average Importance<br>Very Important<br>Absolutely Essential	
I have used the app as a reference under emergent/urgent circumstances:	True/False<br>False	
We would appreciate knowing how the app was useful to you in an emergency:	Free Text Response	
I have used the Society for Pediatric Anesthesia Critical Events Checklist:	True/False	
We would appreciate knowing how the checklist was useful to you:	Free Text Response	
What are is your practice size?	I am the only practitioner for large area<br>One of several practitioners in the area<br>Group practice 1-5 members<br>Group 5-10 members	Group 10-25 members<br>Group 25-50 members<br>Group > 50 members
What is your anesthesia practice model?	Physician only<br>Physician supervised, anesthesiologist on site<br>Physician supervised, non-anesthesiologist physician on site	Physician supervised, no physician on site<br>No physician supervision<br>Not an anesthesia provider
What is your primary practice environment?	Private clinic or office<br>Local health clinic<br>Ambulatory surgery center	Small community hospital<br>Large community hospital<br>Academic department/University hospital
What is community does your practice primarily serve?	Rural<br>Suburban<br>Urban	
I use of the app as a reference for which classes of patients/procedures?	Adult<br>Pediatrics<br>Obstetric<br>Cardiothoracic<br>Intensive Care	Regional<br>Pain<br>Emergency Room<br>Other

Table S2: Survey for collection of information about sugammadex.

Question	Responses	
Q-01 Introduction: In light of the recent approval of sugammadex in the United States for clinical use, we are investigating practice patterns related to the use of this drug globally. This study is for academic purposes and we have no relationship with the manufacturer(s) of sugammadex. This is a 9 question survey and should only take a few minutes to complete. We appreciate your participation.		
Q-02 Do you have access to sugammadex in your clinical practice?	Yes	No, not on formulary
	Yes, but not relevant to my practice	No, not approved in my country
	No or unsure, but not relevant to my practice	
Q-03 How many years have you been using sugammadex?	Range: 0-10	
Q-04 How many times per week would estimate you use sugammadex?	Range: 0-50	
Q-05 Which of the following statements is true?	I know how much it costs my hospital to acquire sugammadex.	Patient charges by individual drug are not applicable to my practice.
	I know how much it costs my patients if I give sugammadex.	I don't know these costs.
	Drug acquisition costs are not applicable to my practice.	I don't know these applicability of these costs.
Q-06 Which of the following best characterizes your use of sugammadex?	(Always) I use it in every case I use nondepolarizing neuromuscular blockade.	
	(Mostly) I use it routinely, instead of classic reversal (neostigmine, pyridostigmine, etc).	
	(Sometimes) I use it in selected cases where alternative classic reversal (neostigmine, pyridostigmine, etc) is inappropriate.	
	(Rarely) I rarely use it.	
	(Never) I never use it.	
Q-07 Which of the following statements are true for your practice?	I have free, unrestricted access to sugammadex.	My access to sugammadex is limited due to policies restricting its use.
	My access to sugammadex is limited due to cost.	None of the above.
	My access to sugammadex is limited due to availability.	
Q-08 Which of the following statements are true for your personal clinical practice?	I limit my use of sugammadex to preserve a limited supply.	I limit my use of sugammadex due to concerns about cost vs benefit.
	I limit my use of sugammadex due to concerns about adverse events.	I limit my use of sugammadex because I am unsure how to use it.
	I limit my use of sugammadex due to concerns about cost.	None of the above.
Q-09 Which of the following adverse events, suspected or proven to be sugammadex related, have you observed in your personal clinical practice?	Anaphylaxis	Incomplete reversal of neuromuscular blockade
	Hypersensitivity	Treatment of rocuronium/vecuronium anaphylaxis
	Bradycardia	Other
	Bronchospasm	None of the above
Q-10 Which of the following adverse events, suspected or proven to be sugammadex related, are you aware have occurred to a colleague in their clinical practice?	Anaphylaxis	Incomplete reversal of neuromuscular blockade
	Hypersensitivity	Treatment of rocuronium/vecuronium anaphylaxis
	Bradycardia	Other
	Bronchospasm	None of the above

Figure S1: Screenshot of the app.

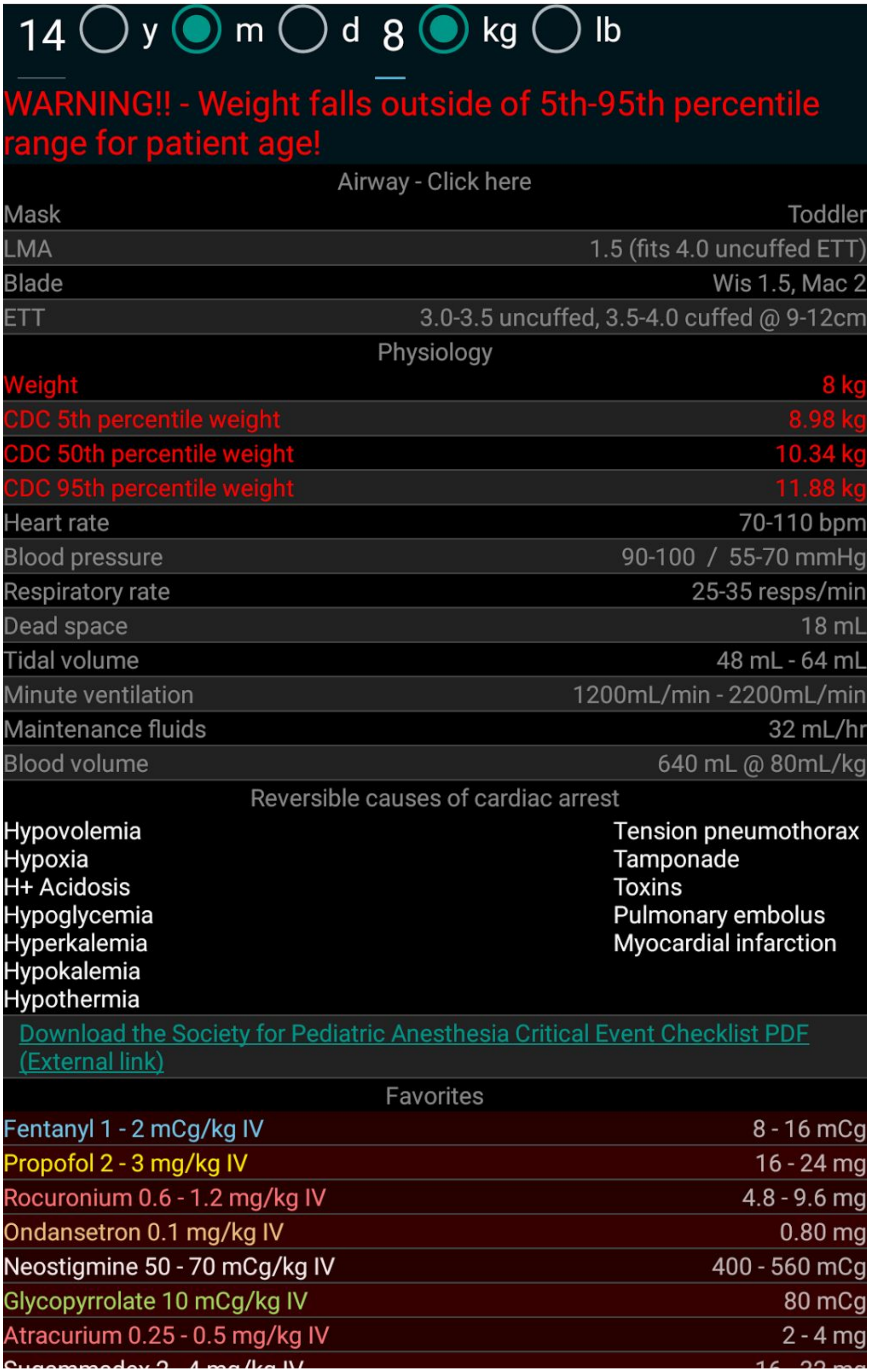

Survalytics Detailed Description

The Survalytics platform is designed to send survey questions to the app and to retrieve survey responses and other analytic metadata from the app. These surveying capabilities are not one-time or static. New survey questions can be delivered via the Internet to the installed base of mobile devices at any time, with the questions being presented to the app users the next time that the app is opened. Survey data and app usage information are transmitted to and from the app utilizing services provided "in the cloud" by Amazon Web Services (Amazon Seattle, WA).

A detailed schema for the survey and analytic data collection was developed. The Survalytics platform allows for the surveys to have a branched structure. Such a branched survey was used to collect basic demographic information from the user after initial installation and agreement by the user to participate in the study. The survey questions are summarized in Table S1. Users had the ability to opt in or opt out of the study at any time.

Location of the device was determined using three different approaches, as described below. For all of the approaches, only the country and "administrative region" were determined and stored, even when more precise determination of location was possible. Here "administrative region" refers to the largest geographical subdivision within the country such as the state in the U.S. or province in India. The precision of the location determination was limited to granularity no more defined than administrative region in order to provide Health Insurance Portability and Accountability Act (HIPAA) compliant de-identification of data. Healthcare providers were entering into the app a patient age and weight. If the location information stored were more precise, patient age and weight information entered into the app might be combined with the

specific location and date in a manner that could potentially comprise protected health information (PHI) as defined by HIPAA.

The first of the three approaches to determining the country and administrative region data was based on GPS coordinates which were reverse geocoded using Google's Geocoding API(Anon n.d.). “Reverse geocoding” refers to the process of converting longitude and latitude coordinates, such as those provided by GPS, into human-interpretable geographic descriptions such as country, state/province, or address. The second approach was based on using the mobile device’s Internet Protocol (IP) address. The IP address was reverse geocoded using a web-based service provided by ip-api.com(Anon n.d.). The last approach was based on the country code stored in the memory chip used to uniquely identify the device (the Subscriber Identity Module or SIM card). Only country information is available via this last approach.

During analysis, the country and administrative region from GPS reverse geocoding was preferentially used. However, GPS coordinates were not always available for a variety of reasons including GPS reception problems, GPS sensor failure, or the device user not consenting to sharing GPS location information. If GPS data were not available, the country and administrative region from IP address was used. Sometimes, this information was not available due to lack of Internet connectivity at the time of data collection. If not, the country from the SIM card (felt to be the least accurate) was used.

The Survalytics platform stores each "event" (e.g. consent, a survey response, an in-app click, or closure of the app) in a local database on the device. When Internet connectivity is detected, one data packet is transmitted from the app at a time, with each packet representing a single "event". Each packet contains relevant details of the event (e.g. what was clicked), as well

as a generic set of information including an anonymous globally unique identifier (generated when the app is first opened on the device), time information (specifically, timestamp, time zone, and local time), location information (from the three sources outlined above), and device language. Transmitted packets are stored as records in an Amazon Web Services DynamoDB database. See the publication describing Survalytics (O'Reilly-Shah and Mackey 2016) for even further additional technical details.

The anonymous user identifier allows for all of the data from one device to be tied together. Together with the time stamps, this allows the sequence of app usage events and survey responses for each mobile device to be reconstructed from the database.

Mobile Healthcare App Study JSON Document Schema

I. Survey/demographics central database tables

The overall architecture is designed to simplify the codebase by using JSON primarily as a transport vehicle and limiting the number of database fields to those that need to be known by the database in question. For example, the AWS source database for downloading questions only needs to know questionguid (for a hash key) and the json_str containing the meat of the question. Telling it ordinal position simplifies other areas of the Android code and so that was included. Otherwise, the content remains unparsed until downloaded by the Android app.

On device, the database is again limited to guid, ordinal position, and jsonstr. The additional fields are flags for internal tracking use. Parsed JSON supplies fields for the generation of the question on-device and for the uploaded response.

http://www.jsoneditoronline.org/
https://www.guidgenerator.com/online-guid-generator.aspx

On AWS: Question Table:

```
questionguid_str       : STRING, PRIMARY HASH KEY
ordinalposition_int    : INT, RANGE KEY
json_str               : STRING
```

json_str JSON Schema: Question

```
{
    surveyname_str          : STRING
    surveyguid_str: STRING
    ordinalposition_int     : INT
    questionguid_str        : STRING
    questionprompt_str      : STRING
    questiontype_str        : STRING
    responses_arr  : ARRAY
        [
            {
            responseid_int          :INTEGER
            response_str            :STRING
            },
            {
            responseid_int          :INTEGER
            response_str            :STRING
            },
            ....
        ]
OPTIONALLY
    conditional_upon_questionguid_str   : STRING // questionguid to check*
```

```
conditional_upon_responseid_int       : INTEGER // responseid to check*
        //*-above two work together and both required to be specified

conditional_upon_datemsid_int         : INTEGER
        // date (in UTC Unix epoch ms) after which to administer this question

conditionalbycountry_str              : STRING // use ISO 3166 alpha-2 codes

delaybydays_int                       : INTEGER
        //wait this many days after the question is first downloaded to ask this question

ongoingquestion_arr                   : ARRAY //array of day of week+time as follows
        [
                {
                notificationtime_str    : STRING
                },
                {
                notificationtime_str    : STRING
                },
                ...
                //notificationtime formatted as follows:  EEEHHmm
                // EEE = three letter day of week (Mon, Tue, Wed, Thu, Fri, Sat, Sun,
Dly)
                //                                         Dly = daily
                // HH = military time hours 00-23
                // mm = minutes 00-59
                //  Examples:      Tue0900, Thu1400, Dly1200
        ]

deletequestion_str                    : STRING //questionguid of ongoing question to
                                                 // delete from local SQLite db

}
```

<u>Local DB on Android</u>

<u>Table questions</u>
questionguid_str
json_str
ordinalposition_int   //Primary key
final_responseid_int
final_response_str
answered_bool
uploaded_bool //unused

Table responses
_id
json
uploaded

II. Responses: Generic schema

The generic schema serves as the basic information passed with all types of uploaded data. The additional overhead is minimal and the presence of this information in each of uploaded packet simplifies future analysis against unnecessary complexity in terms of crossreferences and joins.

```
{
        userguid_str                    : STRING      PRIMARY RANGE INDEX
        localtime_ms_int                : INTEGER     PRIMARY HASH INDEX
        localtime_hrsmilitary_int       : INTEGER
        localtime_dayofweek_str         : STRING
        localtimezone_str               : STRING
        country_tm_str                          : STRING
        lo_lang_str                     : STRING      //locale lang
        app_lang_str                    : STRING
        region_ipapi_str                : STRING      //www.ip-api.com/json
        regionname_ipapi_str            : STRING
        country_ipapi_str               : STRING
        region_gc_str                   : STRING      //geocoding
        country_gc_str                  : STRING
        entrytype_str                   : STRING      LSI // included in all section III items
        ...
}
```

III. Responses: Specific added fields to generic document schema

Survey/demographics data

```
…
entrytype_str          : "survey",
surveyguid_str         : STRING
questionguid_str       : STRING
questionprompt_str     : STRING
response_str           : STRING
responseid_str : STRING //questionguid & "-" Integer.toString(respid)
responses_arr  : ARRAY [if type is multiple response eg checkbox)
        [
                {
                responseid_str          :STRING
                                //questionguid & "-" Integer.toString(respid)
                response_str            :STRING
                },
                {
                responseid_str          :STRING
                                //questionguid & "-" Integer.toString(respid)
                response_str            :STRING
                },
                ....
        ]
```

Consent/Consent Change

```
...
entrytype_str          : "consentcode_int/consentchange_int"
"consentcode_int"      : INTEGER
"consentchange_int"    : INTEGER

                                1 - do not consent
                                2 - consent
                                3 - exit study
                                4 - re-enter study
```

On Start

```
...
entrytype_str                  : "onstart"
"age_yrs_fra"                  : FRACTION
```

```
    "weight_kg_fra"              : FRACTION
```

Age/weight entered by app user (age over 89 to be reported as 89+)

```
    ...
    entrytype_str                : "ageweight",
    "age_yrs_fra"                : FRACTION
    "weight_kg_fra"              : FRACTION
```

Total time using the app

```
    ...
    entrytype_str                : "totaltimeofuse",
    "timeinapp_ms_int"           : INTEGER,
    "ageweightmodified_int       : INTEGER //0=no 1=yes
```

Drugs favorited and changes to favorites

```
    …
    entrytype_str                : "favoriteslist",
    "favoriteslist_arr"          : ARRAY
        [
            {   "drugid_int" : drug.get_id(),          INTEGER
                "name_str": drug.getDrugName(),        STRING
                "position_int" : favepos               INTEGER
            } ,
            {   "drugid_int" : drug.get_id(),          INTEGER
                "name_str": drug.getDrugName(),        STRING
                "position_int" : favepos               INTEGER
            } ,
            ....
        ]
```

In-app clicks (drugs, Epocrates, airway setup guide, critical events checklist, externally linked nerve blocks)

```
    ...
    entrytype_str   :       See the click types below

    Entrytype_str click types:
        "drugclick",
        "epocrates",
        "linkline_str",
        "airwaysetupguide"
```

Extra JSON for drug/epocrates

“drugid_int”	: drug.get_id()
“name_str”	: drug.getDrugName()

Extra JSON for linkline:

“linkline_str”	: STRING == name //nerveblock and spachecklist
“linklineurl_str”	: STRING == link //nerveblock and spachecklist

Methodology for Calculation of App Use Frequency

Under circumstances with no "complications," the frequency of app use for a fixed time interval would be estimated in a straightforward and intuitive manner by counting the number of app uses in the time interval and dividing by the length of the interval. The situation encountered in estimating the app use frequency based on the data obtained from the Survalytics platform is more complicated. This is because the app can be unloaded or otherwise abandoned (e.g., lost phone), and the Android operating system does not allow app unload events to be detected and reported by in-app analytics.

Because of this, estimating the app use rate as the number of uses between the time of consent and the time of conclusion of the study divided by the length of that interval would underestimate, potentially by a large amount, the rate of app use (while the app was available) for any user that unloaded the app or otherwise abandoned it. Similarly, estimating the rate of app use based on a time interval determined by the last time the app was used causes over estimation of the usage rate because the time after the last use until the end of the study (or until the app is unloaded) is truncated from the interval used to calculate the rate.

The approach used here to estimate the usage rates is designed to help correct for these biases in a reasonable way. The method is based on the assumption that, for any user i, the use of the app while installed (or otherwise not abandoned) follows a Poisson distribution with a constant usage rate λ_i. In this case, it can be shown that the expected value of the latest usage time t_n in an interval $[0, \mathrm{T}]$ where there have been n uses in that interval is

$E(t_n) = T\,n/(n+1)$. This last equation is derived from the fact that, for a Poisson process with n events occurring in the time interval $[0,\mathrm{T}]$, the times of those events will have the same distribution as the order statistics of n uniform random variables on the same interval (see, for example, Doob, page. 400) (Doob 1990). The formula above for $E(t_n)$ can be used to estimate T, the end of the time interval. Specifically, the estimated unload time is $\hat{T} = t_n\,(n+1)/n$, where t_n is the latest usage time and n is the number of observed uses.

Using this idea, the usage rate λ_i for user i is estimated as follows. First the app unload time predicted from the time of the last use is estimated by

$$\hat{T}_{U,i} = \frac{n_i+1}{n_i}(t_{n_i} - T_{C,i}) + T_{C,i}$$

where n_i is the number of app uses by user i, t_{n_i} is the time of the last use, and $T_{C,i}$ is the time of consent for user i.

The time which is then used as the end of the time interval in the estimation of the usage rate is the minimum of the estimated unloading time $\hat{T}_{U,i}$ and T_S, the time of the conclusion of the study. The estimate of the rate λ_i for user i is then given by:

$$\hat{\lambda}_i = \frac{n_i}{\min(\hat{T}_{U,i}, T_S) - T_{C,i}} \; .$$

These estimated usage rates will be smaller than ones based on using the last observed time of use, and larger than those based on the end time of the study (unless the estimated unload time is later than the end of the study).

References

Anon. “Google Maps APIs,” [accessed on June 19, 2016 a]. Available at: http://www.webcitation.org/6iO62lCZh.

Anon. “Ip-Api Geolocation API,” [accessed on June 19, 2016 b]. Available at: http://www.webcitation.org/6iO5uZzHT.

Doob, J. L. 1990. Stochastic Processes, Wiley-Interscience.

O’Reilly-Shah, V. and S. Mackey. 2016. “Survalytics: An Open-Source Cloud-Integrated Experience Sampling, Survey, and Analytics and Metadata Collection Module for Android Operating System Apps.” *JMIR mHealth and uHealth*, 4(2): e46 Available at: http://dx.doi.org/10.2196/mhealth.5397, doi:10.2196/mhealth.5397.
